# Evaluation of galectin-3 genetic variants and lipid profile in RA patients in North Indian population

**DOI:** 10.1186/1755-8166-7-S1-P66

**Published:** 2014-01-21

**Authors:** Tarnjeet Kaur, Manpreet Kaur, Jatinder Singh, Sukhdev Singh, Sumeet Arora

**Affiliations:** 1 Department of Human Genetics, Guru Nanak Dev University, Amritsar, India; 2 Department of Molecular Biology and Biochemistry, Guru Nanak Dev University, Amritsar, India; 3 Rheumatology Clinic, Amritsar, India

**Keywords:** Galectin-3, lipid profile, rheumatoid arthritis, SNP

## Background

Rheumatoid arthritis (RA) is a chronic, inflammatory, systemic disease characterized by inflammation and destruction of peripheral joints which leading to deformity and disability. Galectin- 3 is emerging as one of key molecules in pathogenesis of RA. The aim of the present study was to evaluate association of two genetic variants rs4644 and rs4652 of galectin-3 with susceptibility towards RA in North Indian population. The study further involved evaluation of lipid profile variables in cases and controls.

## Methods

The present case-control study involved 200 RA patients diagnosed according to 1987 revised criteria of American college of Rheumatology and 200 unrelated age, sex and ethnicity matched controls. Genomic DNA was isolated from blood samples and genotyping was done with PCR-RFLP. Sample size for genetic association was calculated by CaTS Power calculator (http://www.sph.umich.edu). Serum was analyzed for lipid profile biomarkers using standard reagents and kits. Genotypic distribution of control and RA was compared by odds ratio statistics using medcal software. Differences in lipid profile were analyzed by independent `t’ test using SPSS version 18.0 (IL, USA, and Chicago)

## Results

The genotypic distribution of +191(A/C) showed significant differences between patients and controls (odds ratio = 1.9552, 95% CI = 1.0461-3.6542, p<0.05). AA genotype was found to be more prevalent in patients in comparison to controls. However, genotypic distribution for +292 (C/A) showed no significant difference between controls and cases (odds ratio = 0.2768, 95% CI = 0.0541-1.4149, p>0.05 ). RA patients were found to be dyslipidemic as indicated by the significantly higher atherogenic index as compared to controls (p<0.01).

## Conclusion

Galectin-3 may play an important role in pathogenesis of RA.

